# One Link to Link Them All: Indirect Response Activation Through Stimulus–Stimulus Associations in Contingency Learning

**DOI:** 10.1027/1618-3169/a000597

**Published:** 2024-01-30

**Authors:** Mrudula Arunkumar, Klaus Rothermund, Carina G. Giesen

**Affiliations:** ^1^ Department of General Psychology II, Friedrich Schiller University Jena, Germany; ^2^ Department of Psychology, Health and Medical University Erfurt, Germany

**Keywords:** stimulus–stimulus associations, sensory preconditioning, contingency learning

## Abstract

**Abstract:** A conditioned response to a stimulus can be transferred to an associated stimulus, as seen in sensory preconditioning. In this research paper, we aimed to explore this phenomenon using a stimulus–response contingency learning paradigm using voluntary actions as responses. We conducted two preregistered experiments that explored whether a learned response can be indirectly activated by a stimulus (S1) that was never directly paired with the response itself. Importantly, S1 was previously associated with another stimulus (S2) that was then directly and contingently paired with a response (S2-R contingency). In Experiment 1a, an indirect activation of acquired stimulus–response contingencies was present for audiovisual stimulus pairs wherein the stimulus association resembled a vocabulary learning setup. This result was replicated in Experiment 1b. Additionally, we found that the effect is moderated by having conscious awareness of the S1–S2 association and the S2-R contingency. By demonstrating indirect activation effects for voluntary actions, our findings show that principles of Pavlovian conditioning like sensory preconditioning also apply to contingency learning of stimulus–response relations for operant behavior.


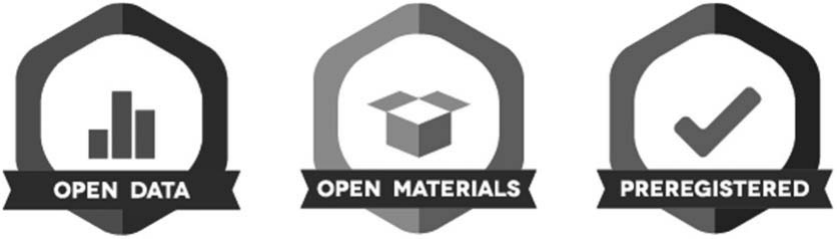


One can pick up associations not only between stimuli and responses but also between two or more stimuli. To demonstrate associations between two stimuli, a combination of stimulus–stimulus (S–S) and stimulus–response (S–R) pairings is used in a procedure called sensory preconditioning (Brogden, 1939) popular in the Pavlovian conditioning literature. In sensory preconditioning, two unrelated, neutral stimuli are repeatedly presented together (e.g., a light and a tone) to create a stimulus–stimulus association in a first phase. Then, in a second phase, one of the stimuli (e.g., light) is paired with an unconditioned stimulus that elicits a response [e.g., food (*unconditioned stimulus*, US) that leads to salivation (*unconditioned response*, UR)]. This renders the light stimulus a *conditioned stimulus* (CS), which elicits salivation as a *conditioned response* (CR; *direct* response activation). In the crucial third and last phase, the other stimulus (i.e., the tone) from the first phase is presented to test whether the *associated stimulus* will also elicit the conditioned response (*indirect* response activation). Evidence for sensory preconditioning (i.e., indirect response activation) has been reported in animals (Espinet et al., 2004; Kimmel, 1977) as well as in humans (Barr et al., 2003; Dunsmoor et al., 2011).

These findings are remarkable because the associated stimulus has never been directly paired with the unconditioned response. Due to its association with the conditioned stimulus (established in the first phase), the associated stimulus can elicit the conditioned response indirectly via an S–S association. In other words, the response can be transferred to another stimulus by means of common associations via the conditioned stimulus.

Recent studies show that such transfer effects also occur in human learning, evidenced in both neurological (Wimmer & Shohamy, 2012) and behavioral studies (e.g., Bejjani et al., 2018). Studies on evaluative conditioning also demonstrate learning effects that are reminiscent of sensory preconditioning (Walther, 2002). Walther (2002) showed that the spreading attitude effect to another stimulus that was not directly paired with a valence value occurred even without explicit verbal knowledge or awareness of the associations (see also De Houwer et al., 2001; Hammerl & Grabitz, 1996). Beyond attitudes, the semantic meaning of words is also transferrable to similar words (e.g., synonyms) or to pseudowords that co-occurred with a meaningful word (Staats, Staats, & Heard, 1959; Staats, Staats, Heard, & Nims, 1959). Pavlovian conditioning (PC) effects typically occur at the level of reflexes (i.e., autonomous responses to biologically relevant stimuli). Against this background, sensory preconditioning is an interesting phenomenon because it reflects a learning effect for stimuli without biological relevance. Although sensory preconditioning-like effects have been explored to show transfer of learning in these above examples with attitudes (Staats & Staats, 1958) and semantic meaning, it has not yet been directly tested with voluntary responses. It is also striking that in terms of procedure and also in terms of effects, many PC principles known from animal studies can be transferred to contingency learning in humans (for an overview, see De Houwer & Beckers, 2002). Hence, we explored whether sensory preconditioning-like effects are possible in human contingency learning. Demonstrating such an effect in the contingency learning paradigm will foster our understanding of the processes underlying human contingency learning. In particular, it will shed light on the question whether PC principles also apply to recency-based episodic retrieval processes to which contingency learning effects have been attributed (C. G. Giesen et al., 2020; Schmidt et al., 2020).

## Study Aims and Hypotheses

In the present study, we aim to investigate the phenomenon of sensory preconditioning in a contingency learning (CL) paradigm with voluntary actions (Schmidt et al., 2007). In this paradigm, Schmidt et al. (2007) systematically paired words with colors and responses in a color classification task, which facilitates responding (faster responses, less errors) for frequent word–color combinations compared to rare combinations (Schmidt et al., 2007; see also Schmidt & De Houwer, 2019). This paradigm is structurally similar to a PC paradigm in that irrelevant stimuli (words, ≈CS) are systematically paired with relevant stimuli (colors, ≈US) and responses (key presses, ≈UR), eventually leading to an activation of response tendencies (≈CR) that are related to the contingent color for the previously neutral word stimuli. The crucial difference between this type of CL and a prototypical PC paradigm is the type of the response. Whereas PC studies typically focus on respondent behavior, that is, on responses that are unconditionally triggered by certain stimuli (e.g., saliva secretion elicited by food; reflexes), the CL paradigm investigates the transfer of a voluntary response (e.g., key press) that is assigned to an eliciting stimulus via arbitrary task rules (e.g., blue font color → press left) to an irrelevant stimulus that is contingently paired with the relevant stimulus or response. Due to the structural similarity between the two paradigms, it has been speculated that CL effects might be driven by similar mechanisms as PC effects (e.g., C. Giesen & Rothermund, 2014) and thus should be subject to the same principles that have already been demonstrated in the realm of PC (e.g., *overshadowing*; Arunkumar et al., 2022). To further test this hypothesis, we conducted a series of experiments that investigated whether sensory preconditioning effects that have regularly been demonstrated in PC can also be obtained for human contingency learning since CL and PC share structural similarities (De Houwer & Beckers, 2002). Regarding previous learning studies, evidence for such a transfer was observed using cognitive control states: Bejjani et al. (2018); valence information: Walther (2002); or motivationally incentivized choices: Wimmer and Shohamy (2012). In the present study, we investigated whether learnt stimulus–response contingencies can be transferred to an associated stimulus, which would demonstrate indirect response activation effects in contingency learning involving voluntary responses. Specifically, we explored whether multimodal stimulus pairs can foster such an indirect response activation effect of the learnt contingent response to the associated stimulus from another modality.

As commonly seen in everyday life, we are exposed to stimuli from different modalities. Particularly in language learning, we pick up vocabulary from both audio and visual cues and associate it with the word we know in our native language. As already found in the literature, semantic properties of the words can also be transferred to associated stimuli that are other words or pseudowords (e.g., Staats, Staats, & Heard, 1959). Language learning models have also explored the mechanism underlying how we learn new foreign language words, hypothesizing that the association between words (for example, foreign language word and native language words) mediated the association of the new word and the referent object (e.g., Dijkstra & Van Heuven, 2002; Kroll et al., 2010). Through this indirect lexical access, one can deduce meaning and learn new vocabulary. Inspired from this rationale, we created a paradigm that resembled a vocabulary learning scenario to investigate whether two newly associated stimuli can transfer learnt responses from one stimulus to the other.

Our contingency learning paradigm works as follows: In Phase 1, two unrelated stimuli (a made-up language new word (pseudoword) and a German word) are presented together. The pseudoword is always presented auditorily, whereas the German word is presented visually on screen. Participants are instructed to observe and read the German word aloud, which should help in learning stimulus–stimulus (S1–S2) associations. This was later tested at the end of the experiment using a cued recall test. We chose a pseudoword as an auditory stimulus to resemble a new vocabulary learning setup. In Phase 2, stimulus–response (S–R) associations for one stimulus (e.g., S2) of each S1–S2 pair were established by presenting the S2 word as a contingent predictor for a number identification response in Phase 2. In Phase 3, we tested whether the *other* associated stimulus of each pair (i.e., S1) can access and indirectly activate the response that was linked to its associated stimulus (S2) in the preceding Phase 2.

To test whether S1 stimuli can trigger indirect response activation that is mediated by an associated stimulus, a free choice paradigm was chosen. In a free choice task, participants can freely choose which action to perform (typically, key presses) to a presented stimulus. Such a task is commonly used to examine which cognitive mechanisms underlie the production of voluntary actions (Elsner & Hommel, 2001; Vogel et al., 2018). This presents a viable method to investigate whether a stimulus can access and indirectly activate the response that was linked to its associated stimulus.

Indeed, many principles from PC known from animal studies can be transferred to human contingency learning at the level of voluntary responses (for an overview, see De Houwer & Beckers, 2002). However, obtaining PC effects in humans typically requires explicit awareness of stimulus pairings in participants (De Houwer, 2009; Lovibond & Shanks, 2002; Mitchell et al., 2009), whereas S–R contingency learning can be acquired (Schmidt et al., 2007, 2010) and also retrieved (C. Giesen & Rothermund, 2015) independent of awareness. It is thus not clear whether contingency learning in more complex learning setups such as sensory preconditioning requires awareness of stimulus pairings. To investigate any mediating role of awareness for indirect response activation effects, we added measures of S–S and S–R awareness.

## Experiments 1a and 1b

Two experiments were designed to test indirect response activation by accessing learnt S–R contingencies for previously associated stimuli. Experiment 1a aimed to establish a connection between two stimuli of different modalities (i.e., a familiar German word presented visually and a new pseudoword presented auditorily). We then aimed to test whether a learnt stimulus–response contingency for the German word can then later be accessed by the associated pseudoword and affect free choices in a guessing task. Furthermore, we replicated Experiment 1a in Experiment 1b to further validate the findings of Experiment 1a by counterbalancing the stimulus pairs and contingencies across participants to eliminate the potential confound of type of stimuli and responses in leading to an indirect response activation. All materials, preregistrations, data, and analyses for all experiments are available online (https://osf.io/aj2eg/).

### Method

#### Required Sample Size and Preregistration

The sample size was determined based on a priori power calculation using G*Power (Faul et al., 2007). To detect an effect of *d*_*z*_ = .40 (Brysbaert, 2019) with a power of 1 − β = .80 and α = .05, *N* = 71 participants were needed. The study design and analyses plan for Experiment 1a and Experiment 1b were preregistered on the Open Science Foundation (OSF) using the AsPredicted.com template (https://doi.org/10.17605/OSF.IO/FC5U3 for Experiment 1a and https://doi.org/10.17605/OSF.IO/TCRM5 for Experiment 1b).

### Participants

In Experiment 1a, *N* = 71 participants were recruited (*M*_age_ = 21.76 years). The experiment was an online study, built on PsychoPy (v2021.2.3; Peirce et al., 2019), and was hosted on Pavlovia (https://pavlovia.org/) for online data collection and lasted for 20 min. Participants were German students of FSU Jena and other participants in the age range of 18–35 years who were recruited through word of mouth. Among the participants, those who were students of FSU Jena were compensated with partial course credits. For Experiment 1b, *N* = 71 participants were also recruited (*M*_age_ = 21.14 years); however, this time the participants were recruited via Prolific and were German native speakers between the age group of 18–35 years. The participants were compensated £ 3.50 according to the norms of Prolific. Only German native speakers were recruited since the stimulus pairs used in both Experiment 1a and Experiment 1b had a German word as S2. Informed consent was given by the participant at the start of study by pressing “j” upon reading the form displaying the details of the study, the type of data collected, compensation amount, and if there are any known risks in participating in this study. Ethical approval was not required for this study as we did not convey any misleading or suggestive information (this is in accordance with the ethical standards at the Institute of Psychology of FSU Jena).

### Material and Procedure

**Figure 1:**
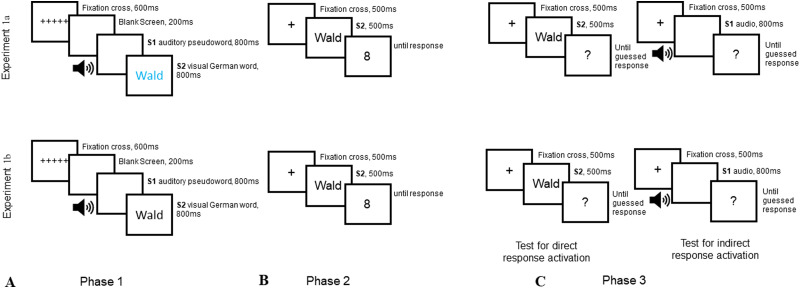
Illustration of the trial sequence for the (A) S–S association phase, (B) forced choice trials in Phase 2, and (C) free choice trials in Phase 3 for both the experiments. “Wald” is the German word for forest that was used as a stimulus since the participants were German native speakers. For illustrative purposes, the text that is not colored is displayed in black with a white background; however, the experiment had a black screen with the text displayed in color in Experiment 1a and white in Experiment 1b.

The participants were instructed to only use their laptop. This study consisted of three phases. Before each phase, the instructions were displayed in white font on a black screen. In the first phase, participants were made to learn a stimulus–stimulus association wherein the visual S2 always followed a particular auditory S1 (100% contingency). Two S1–S2 pairs were introduced in Phase 1 of the study. The participants were asked to read the S2 aloud and informed that the responses would be recorded by the microphone. To make this more convincing, prior to Phase 1, participants also read a short question that tested the microphone, and few reminders were also provided to read the word aloud. However, no microphone response was recorded, nor was there any access to their microphones. We used this mock setup to ensure that the participants paid attention to the word pairs during Phase 1 (this was revealed to participants when they were debriefed at the end of the study). The stimuli were chosen to be tailored for German participants. As S1, the pseudowords were chosen from a list of existing pseudowords (Simone et al., 2020) that were standardized and checked for being phonotactically legal with German. *Mank* and *dels* were the selected pseudowords (S1) that were recorded by a female German native speaker. As S2, *Haus* (house) and *Wald* (forest) were selected as the German words. The screen was black, and the words were presented in the Arial font with height 0.04 (units of PsychoPy). In Experiment 1a, to add a layer of distinction between the audiovisual displays and the pairs, the words were displayed in color: *Haus* was shown in blue, and *Wald* in yellow. The color was irrelevant to the task or the study design and was not mentioned in the instructions. However, since this did not serve any purpose, in Experiment 1b, the words were displayed in white against a black background. In Experiment 1a, all participants observed *mank* followed by *Haus* and *dels* followed by *Wald*, and this was not counterbalanced across participants. To eliminate any confound of a stimulus pair favoring response transfer, we counterbalanced the stimulus pairs in Experiment 1b such that approximately half of the participants (*N* = 38) learnt the pair of *mank* (S1) – Haus (S2) and *dels* (S1) – Wald (S2) and the rest of the participants (*N* = 33) learnt the pair of *mank* (S1) – Wald (S2) and *dels* (S1) – Haus (S2). In total, Phase 1 consisted of 80 trials (40 occurrences of each pair). The association between the pseudoword and German word was built using a 100% contingency. A given trial started with a row of fixation crosses displayed for 600 ms followed by a blank screen for 200 ms and an auditory presentation of pseudowords for 800 ms followed by the visual presentation of the German word for 800 ms (see Figure 1). To establish a S2-R contingency, a forced choice number identification task was used in Phase 2. Participants had a short attention check to see if they remembered the instructions accurately. After the attention check, there was a short practice block consisting of eight trials after which Phase 2 began. Participants saw the number 4 or 8 that appeared in the middle of the screen and responded by pressing the corresponding number key on the keyboard. The S2 (German visual word) was predictive of the number keypress with a 90% contingency. In Experiment 1a, 90% of the time, *Haus* was followed by the number 8 and *Wald* was followed by the number 4 for all the participants. In Experiment 1b, stimulus–response assignment in Phase 2 was also counterbalanced: For half of the participants (*N* = 35), *Haus* was mostly predictive of the number (thus response key) 8 and *Wald* was mostly predictive of 4, both with a 90% contingency. The remaining participants (*N* = 36) observed a 90% contingency of *Haus* followed by the number 4 and *Wald* followed by the number 8. The trials where the contingent number was shown are referred to as valid trials, and the trials where the noncontingent number appeared are referred to as invalid trials. Phase 2 consisted of 100 trials (90 valid trials and 10 invalid trials). The trial sequence in both the experiments (see Figure 1) was as follows: First, a fixation cross was displayed for 500 ms and the S2 was displayed for a fixed amount of 500 ms followed by the number 4 or 8 presented in the center of the screen until the response was given. Participants received error feedback and were asked to press the correct key, and they were warned if they took longer than 2,000 ms to respond.

Phase 3 contained only free choice trials where participants guessed what number they expected to appear after a particular word. S1 or S2 words could appear in Phase 3. Participants were informed of the accuracy rate for S2 guesses in the free choice trials at the end of Phase 3. In total, this phase consisted of 80 trials, 40 with S1 and 40 with S2. The trial sequence was similar to Phase 2, wherein after the display of fixation cross for 500 ms, either S2 words were again presented visually for 500 ms or S1 pseudowords were presented auditorily for 800 ms (which was the length at which the audio words could be heard clearly). Both, words (S2) and pseudowords (S1), were followed by “?”, and we asked participants to freely choose the response by pressing the relevant response key depending on the number they guessed should have appeared (Figure 1).

After Phase 3, a short cued recall test regarding the S1–S2 pairs and a questionnaire followed. This test consisted of two trials where each trial started with a fixation cross for 500 ms followed by S1 for 800 ms. After this, a “?” appeared for 800 ms followed by a question asking what word should have appeared with three options: One option was the correct associated S2 and the other two options were the other remaining S2 word and “do not know,” the order in which the options were presented on screen was randomly generated for each stimulus. We asked the participants to press the number corresponding to the option containing the correct associated S2. For Experiment 1b, only two options were shown, as the “do not know” option was removed. After the cued recall test, a questionnaire in German followed where we asked questions concerning their level of concentration and whether they had the impression that they learnt a new language. The questions (translated) were as follows: “During the study, did you have any distractions?” and “Did you learn a word from a new language?”, which could have meant that they transferred the semantic meaning of the German word that followed the pseudoword. We instructed the participants to respond in a forced choice yes/no manner where they were asked to press “j” if yes, “n” if no, and “k” if they are unsure or do not know. Additionally, we assessed awareness of S2-R contingency also in the form of a questionnaire. The questions (translated) were as follows: “What number mostly occurred with Haus/Wald?” The question was presented on the screen, and participants were asked to respond by pressing the key, 4 if the response is 4, 8 if the response is 8, and “k” if they do not know. Finally, questions regarding the possible response guess for S1 were also presented: “What number do you think could have occurred with mank/dels” (presented auditorily; response options: 4, 8, k for do not know)? In Experiment 1b, the do not know option was removed to have a more direct measure of awareness.

### Design

In Phase 2, contingency learning between S2-R contingencies was analyzed by comparing the performance (in reaction time and error rates) in valid (90%) and invalid (10%) trials. In Phase 3, the performance was assessed by measuring the proportion of response choices that corresponded to valid contingent responses. Hence, for S2 words, free choice performance served as an additional check for contingency learning (direct response activation). To test the hypothesis, the performance of S1 free choice trials (S1 Transfer) was analyzed to check whether participants transferred the valid contingent response of the associated visual S2 to auditory S1, thus assessing the indirect response activation effects.

### Data Analysis

We used R (version 4.1.2; R Core Team, 2022) for all our analyses, namely packages stats (v4.2.1) for the analysis concerning the direct and indirect retrieval effects and lme4 for the analyses using the multilevel modeling to assess the role of awareness.

## Experiment 1a Results

### Data Preparation

All participants were included in the analyses. No data were collected from Phase 1 (however, memory for S–S associations was assessed at the end of the experiment). Reaction time (RT) and error rates (ER) were collected for Phase 2. For RT analyses, erroneous RTs (5.3%) and RT outlier^1^ values per individual (3.6%) were excluded from all analyses. Response choices (%) were collected for Phase 3.

### Contingency Learning Effects

#### Phase 2 (Acquisition of S–R Contingencies)

For the forced choice number identification task, the RTs and ERs were analyzed as a function of validity (valid vs. invalid). Table 1 shows the mean RT per validity condition. For RT, a directional *t* test revealed that participants performed significantly faster on valid compared to invalid trials, Δ = 30.4 ms, *t*(70) = 6.31, *p* < .001, *d*_*z*_ = 0.75. The same was true for ER, as participants committed less errors for valid compared to invalid trials, Δ = 13.7%, *t*(70) = 6.31, *p* < .001, *d*_*z*_ = 0.75 (Table 1). This indicates that participants successfully learnt the association between the S2 and the response and exhibited S2-R contingency learning.

**Table 1:** Mean reaction time and error rate (*SD*) of the performance of trials containing S2 stimuli in Phase 2 for both the studies

Experiment	Reaction time (in ms)			Error rate (in %)		
	S2-R			S2-R		
	Valid	Invalid	CL effect	Valid	Invalid	CL effect
Experiment 1a	418 (53)	448 (56)	30	3.9 (2.9)	17.7 (17.9)	13.7
Experiment 1b	411 (49)	434 (53)	23	3.3 (3.3)	11 (12.9)	7.6
*Note*. CL effect = contingency learning effect computed as mean of invalid trials – mean of valid trials.						

#### Phase 3 (Direct Response Activation of Acquired S–R Contingencies)

**Figure 2:**
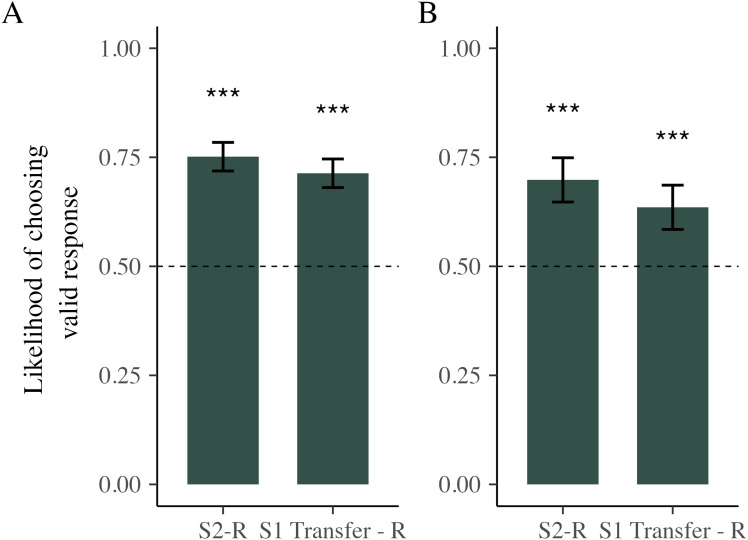
(A) Mean proportion of valid keypresses per stimulus type in Phase 3 in Experiment 1a. (B) Mean proportion of valid keypresses per stimulus type in Phase 3 for Experiment 1b. 50% of mean proportion of valid responses indicates the chance level of choosing the valid response. Error bars: ± CI, ***p < .001. **p < .05.

To check the response activation effects, we analyzed the proportion of valid response choices for S2 words. If the response choice was the response that corresponded to the S2-response mapping from Phase 2, it was labeled as a valid response choice. If the response chosen reflected the other, noncontingent response, then it was labeled as an invalid response choice. For the S2s, the participants’ proportion of valid response choices was tested against 50% to check whether they more often chose the contingent response, thus providing additional evidence showing that S2-R contingency was established. The directional *t* test results showed that the mean proportion of valid response choices for S2 was significantly better than 50%, Δ = 75.1%, *t*(70) = 7.29, *p* < .001, *d*_*z*_ = 0.87 (see Figure 2).

### Indirect Response Activation Effects

To test whether participants were able to transfer the response from the associated S2 to an S1 that was never directly paired with the response, the free choice responses for S1 Transfer stimuli were analyzed. For S1, response choices that corresponded to the associated S2-response mapping from Phase 2 were coded as valid response choices; otherwise, they reflected invalid response choices. While looking at the performance for the auditory S1 trials, the participants also chose valid responses significantly more often than chance level (50%), Δ = 71.3%, *t*(70) = 6.62, *p* < .001, *d*_*z*_ = 0.79. As an exploratory analysis suggested by an anonymous reviewer, we found that these indirect activation effects did not significantly differ from the direct activation effects using a paired *t* test, *t*(70) = 1.63, *p* = .10, *d*_*z*_ = 0.19. This supports the evidence that participants can transfer the response even across modalities from a native language word in visual modality (S2) to an associated pseudoword in an auditory modality (S1 Transfer; cf. Figure 2).

### Role of Awareness

**Figure 3:**
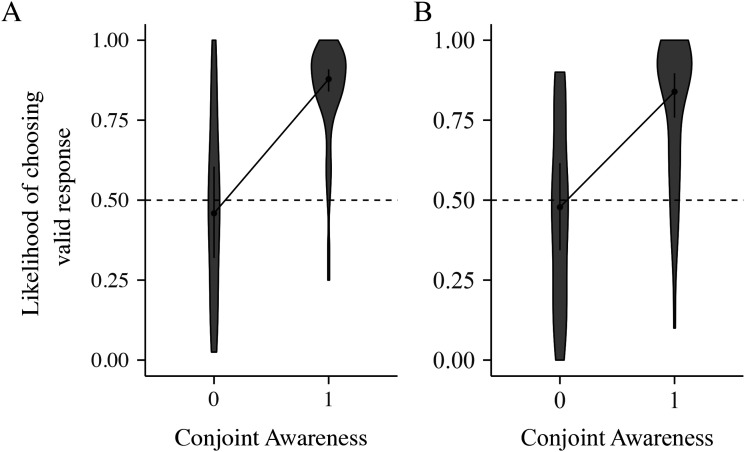
Plot from the model including the factor of awareness of both SS association and S2-R Contingency for (A) Experiment 1a and (B) Experiment 1b. Having awareness of both S1–S2 pairs and S2-R contingency was associated with a higher chance of choosing the valid response for a respective S1 Transfer stimulus in both experiments.

We also explored the role of participants’ conjoint awareness of S1–S2 and S2-R contingencies for both S1–S2 pairs. Table 2 shows the number of participants per raw accuracy score level for the questions that explicitly asked about the stimulus–response contingencies for S2 stimuli (two questions, i.e., one for each S2 stimulus) as well as accuracy scores from the cued recall test assessing memory of S1–S2 associations (two questions, i.e., one for each S1 stimulus). To assess the role of awareness, we created a composite awareness score that coded for each S1 Transfer stimulus whether participants had awareness of *both*: the S1–S2 association and the S2-R contingency relation of the associated S2. Note that this predictor can take a value of 0 (indicating that participants had no conjoint awareness of S1–S2 and S2-R contingencies for this S1) or 1 (indicating that participants correctly identified both, S1–S2 and S2-R contingencies for this S1). A score of 0.5 indicates that the participants were aware of either the S1–S2 association or the S2-R contingency (see Table 2). For the analysis of the role of awareness, only the values of 0 and 1 per stimulus were considered. This composite awareness score was then entered into a multilevel random intercept model on proportion of valid response choices for S1 Transfer stimuli to test the role of having awareness of both S1–S2 association and the S2-R contingency on choosing the valid response for the S1 Transfer stimuli in Phase 3. The model showed a significant role of awareness in producing the effects of indirect response activation (OR = 8.52, *p* < .001; see Table 3). Being aware of both S1–S2 and S2-R relations made participants eight times more likely to produce a valid response choice for the S1 Transfer stimuli. It showed that the indirect response activation effects are mediated by conjoint awareness of both the S1–S2 association and the S2-R contingency (Figure 3).

**Table 2:** Number of participants per accuracy score level (raw scores) based on the cued recall test (assessing S1–S2 associations) and the S2-R contingency question presented at the end of each experiment

Raw Accuracy Score	Experiment 1a, number of participants		Experiment 1b, number of participants	
	S–S	S2-R	S–S	S2-R
0	15	19	9	23
1	0	2	11	0
2	56	50	50	47

Composite Awareness Score^a^	Number (%) of participants	Number (%) of participants
Composite score indicating awareness of both S1–S2 and S2-R across stimulus pairs		
0	20 (28.2%)	27 (38.6%)
0.5	8 (11.2%)	10 (14.3%)
1	43 (60.6%)	33 (47.1%)
*Note*. Based on this raw accuracy scores, we computed composite awareness scores and provide the number of participants who had awareness for both S–S and S2-R contingencies.							
^a^A composite awareness score of 1 indicates that participants had both S1–S2 and S2-R awareness for both experimental S1–S2 pairs; a score of 0.5 indicates that participants had S1–S2 and S2-R awareness for only one experimental S1–S2 pair; and a score of 0 indicates that participants were not conjointly aware of both S1–S2 and S2-R contingencies.							

**Table 3:** Multilevel analysis on proportion of valid response choices for S1 Transfer stimuli in Phase 3 as a function of having awareness of both the S1–S2 relation and the S2-R contingency relation for a given S1 (1 = conjoint awareness, 0 = no awareness; Level 1 predictor)

Effects	Experiment 1a			Experiment 1b		
	OR	*SE*	Statistic	OR	*SE*	Statistic
Intercept	0.85	0.26	−0.55	0.92	0.26	−0.31
Awareness of SS and S2R	8.52	3.01	6.07***	5.70	2.36	4.20***
Model fit						
*Note*. ***p < .001.						

## Experiment 1b Results

### Data Preparation

The same exclusion criteria for slow, fast, and incorrect RTs as for Experiment 1a were implemented in Experiment 1b. Due to excessive error rates (100%), data of one participant were excluded from all the analyses. Thus, we proceeded with *N* = 70 participants. Accordingly, at the trial level, for RT analyses in Phase 2, erroneous trials (4.1%) and RT outlier values per individual (4%) were excluded.

### Contingency Learning Effects

#### Phase 2 (Acquisition of S–R Contingencies)

For the forced choice number identification task, the RTs and ERs were analyzed. S2-R contingency learning was tested as a function of validity (valid vs. invalid). For RT, participants performed significantly faster on valid compared to invalid trials, Δ = 22.8 ms, *t*(69) = 5.38, *p* < .001, *d*_*z*_ = 0.64. The same was true for ER, as participants committed less errors for valid compared to invalid trials, Δ = 7.6%, *t*(69) = 4.9, *p* < .001, *d*_*z*_ = 0.59 (Table 1). This indicates that participants successfully learnt the association between the S2 and the response and exhibit successful S2-R contingency learning.

#### Phase 3 (Direct Response Activation of Acquired S–R Contingencies)

Similar to Experiment 1a, we analyzed the proportion of valid response choices made for the free choice S2 trials in Phase 3. For S2s, the participants’ proportion of valid response choices was tested against 50% to check whether they were inclined to choose the contingent response. The *t* test results showed that the mean proportion of valid response choices for S2 was significantly better than 50%, proportion of valid responses Δ = 69.8%, *t*(69) = 5.15, *p* < .001, *d*_*z*_ = 0.62 (see Figure 2).

### Indirect Response Activation Effects

While looking at the performance for the auditory S1 Transfer trials, the participants also chose valid responses significantly more often than chance level (50%), Δ = 63.5%, *t*(69) = 3.76, *p* < .001, *d*_*z*_ = 0.45. Similar to Experiment 1a, we found that these indirect activation effects did not significantly differ from the direct activation effects using a paired *t* test, *t*(69) = 1.74, *p* = .08, *d*_*z*_ = 0.20. This further validates the result that participants can transfer the response even across modalities from a native language word in visual modality (S2) to an associated pseudoword in an auditory modality (S1 Transfer; cf. Figure 2).

### Role of Awareness

The accuracy scores for S1–S2 and S2-R relations at the end of the experiment were calculated (cf. Table 2). The composite score referring to the participants’ conjoint awareness of the S1–S2 association and the S2-R contingency for each auditory S1 was computed. This composite awareness score for the particular stimulus (only 0 and 1) was then entered into a multilevel random intercept model using the proportion of valid response choices for S1 Transfer stimuli as a dependent variable to test the role of having awareness of both S1–S2 association and the S2-R contingency on choosing the valid response for the auditory S1 in Phase 3. The model showed a significant role of awareness in producing the effects of indirect response activation (OR = 5.70, *p* < .001; see Table 3) where the combined awareness of *both* S1–S2 association and the S2-R contingency made participants five times more likely to choose the valid response choice. Thus, this finding adds further support for the evidence that the indirect response activation effects are mediated by conjoint awareness of both the S1–S2 association and the S2-R contingency (Figure 3).

## General Discussion

We conducted two experiments^2^ to explore whether a voluntary response can be indirectly activated by a stimulus (S1) that was never directly paired with the response itself. Crucially, S1 was previously associated with another stimulus (S2) that was directly and contingently paired with a response (S2-R contingency). A similar phenomenon has been demonstrated in animal and human PC studies using the sensory preconditioning paradigm. Our study aimed to look at whether such a transfer is possible in a contingency learning paradigm (Schmidt et al., 2007) that uses operant behavior – i.e., behavior that is under voluntary control. We therefore employed a contingency learning paradigm (Schmidt et al., 2007) to contingently pair a voluntary response with a stimulus and later test if it can be indirectly activated by an associated stimulus that had previously been paired only with the first stimulus (indirect transfer). Notably, we used multimodal stimulus pairs resembling a vocabulary learning setup involving an auditory pseudoword (new language word) and a native language word (presented visually) as the S1–S2 association. Both our experiments found that indirect response activation effects were present, indicating that the auditory S1 could indirectly activate the response that was contingently paired with the associated visual S2. Our results show that sensory preconditioning-like effects can be demonstrated at the level of human contingency learning using voluntary responses.

Although we obtained reliable and robust effects of indirect response activation in both experiments, we want to point out that this might not always be the case (see also footnote 2). Thus, one could argue that indirect response activation effects are limited to conditions in which S–S pairs are particularly intuitive to learn. The present experiments endorsed a setup that resembled vocabulary learning, which could have made it easier for participants to remember the S1–S2 association. Possibly, a form of semantic generalization occurred, meaning that pseudowords were assumed to share semantic features with the German words. This might have aided memory for S1–S2 associations and indirect response activation (Staats, Staats, & Heard, 1959) and further supports the claim that the intuitiveness of the stimulus pairs can contribute to indirect response activation effects. Alternatively, the multimodality of S1–S2 pairs in Experiments 1a and 1b could have enhanced the encoding of the word pairs, which would also result in better memory for S1–S2 associations as seen in the accuracy scores during the cued recall test and thus large indirect response activation effects. Together, semantic generalization and/or multimodality of the stimuli could have been beneficial for the emergence of indirect response activation effects, which supports the idea that the type of S1–S2 association can have an influence on how successfully responses can be indirectly activated and transferred to the associated stimulus (Baeyens et al., 1993; Todrank et al., 1995).

Along similar lines, we also found that awareness played a prominent role in Experiments 1a and 1b. Here, the indirect response activation effects were mediated by the conjoint awareness of both the S1–S2 association and the S2-R contingency. Since there was a high number of participants with conjoint awareness in Experiment 1a (*N* = 43, reflecting 61% of the sample) and in Experiment 1b (*N* = 33, 47% of the sample; Table 2), it could account for the presence of larger indirect response activation effects. This is a noteworthy finding because it suggests that indirect response activation effects can follow from contingency awareness (cf. De Houwer, 2009) rather than automatic activation of stimulus–stimulus and/or stimulus–response associations (C. Giesen & Rothermund, 2015; Schmidt et al., 2007, 2010). Whereas studies on the spreading attitude effect show that transfer can occur without having conscious access to these relations (Baeyens et al., 1993; Walther, 2002), this seems not to be the case for human contingency learning in more complex learning setups. Therefore, our findings also contribute to the knowledge of factors such as awareness that are conducive to a successful response transfer to an associated stimulus at least under specific conditions.

### Implications

Several aspects are noteworthy about the present findings. First, although there are studies demonstrating sensory preconditioning-like effects on a behavioral and neurological level (Bejjani et al., 2018; Wimmer & Shohamy, 2012), the present study presents the first evidence for indirect response activation in human contingency learning with instrumental responses. Responses in our study were simple key presses with no history of reward (cf. Wimmer & Shohamy, 2012) or evaluative meaning (Walther, 2002). Second, the findings of our study point toward a strong modulatory influence of awareness (regarding underlying stimulus–stimulus and/or stimulus–response relations) on indirect response activation for voluntary controlled responses. Further evidence on similar influences of contingency awareness on contingency learning in more complex learning setups comes from previous studies that explored overshadowing-like effects (Arunkumar et al., 2022) and evaluative learning effects (C. G. Giesen et al., in prep) in contingency learning tasks. On the one hand, this insight is consistent with the claim that Pavlovian conditioning effects in humans require explicit awareness of pairings (e.g., De Houwer, 2009; Lovibond & Shanks, 2002; Mitchell et al., 2009). On the other hand, this finding contrasts with previous explanations of contingency learning as being automatic, reflecting retrieval of incidental and transient stimulus–response bindings that do not require awareness (C. G. Giesen et al., 2020; Schmidt et al., 2020; see also Jiménez et al., 2022; Rothermund et al., 2022; Xu & Mordkoff, 2020). Dissociating the roles of awareness-mediated learning and learning that is due to (direct or indirect) stimulus-based retrieval processes may therefore be a promising avenue for future research. Third, there are several potential explanations with regard to the mechanisms underlying the present findings. According to one view, it could be that participants first form S1–S2 associations (Phase 1) and S2-R associations (Phase 2) independently of each other. Presenting S1 alone (Phase 3) will then first activate the associated S2, which will then activate the associated R (chain learning model). However, other scenarios are possible. For instance, it could be that repetition of the S2 in Phase 2 will activate the associated S1, which will then directly become associated with the response to S2 (mediated learning model^3^). Note that both accounts can explain the findings of the present experiments. We want to point out that our major research aim was to demonstrate that in principle, sensory-preconditioning-like effects are possible in human contingency learning. The present experiments were not designed to dissociate between both learning models. In our view, dissociating possible underlying mechanisms behind the basic indirect response activation effect is a promising endeavor for future research. Fourth, as shown in Experiments 1a and 1b, the design of the experiment intended to replicate a scenario where we might learn a foreign language by experiencing mere occurrence of the new word with a word from a native language. In this case, the co-occurrence of the foreign language word and native language word form an association, which could have been further strengthened by the multimodality feature of the words and/or the intuitiveness of semantic features of the native language words. Later, the appropriate behavior learnt for the native language word is transferred to the foreign language word, which could be reflected in ascribing a shared semantic meaning or an action, like stopping when you see the “stop” sign in a new language. Most importantly, this can occur without having an explicit learning instruction. It can arise from making spontaneous inferences based on stimulus–stimulus and/or stimulus–response co-occurrences that occur in everyday life. Thus, the finding proves useful in aiding vocabulary learning indirectly where the semantic information is transferred. Future research can aim to explore whether this is enhanced and speeds up the language learning process when it is explicitly mentioned that the stimuli associations have the same meaning. Moreover, based on the glimpses from our preliminary data, closely examining the extent of these transfer effects based on the type of stimulus associations can also be an interesting avenue for future research.

### Conclusion

We employed the sensory preconditioning paradigm to assess indirect response activation effects in human contingency learning. In detail, we investigated whether a learned response can be indirectly activated by a stimulus (S1) that was never directly paired with the response itself. Importantly, S1 was previously associated with another stimulus (S2) that was then directly and contingently paired with a response (S2-R contingency). Our findings support that indirect response activation effects, which are reminiscent of sensory preconditioning, emerge even within a contingency learning task. This is present when the context is suggestive of a language learning scenario and consists of multimodal stimuli associations. Importantly, indirect response activation effects for S1 are mediated by and therefore due to having conjoint awareness of both the S1–S2 and S2-R contingencies.

## Footnotes

^1^
RT faster than 150 ms or slower than 1.5 interquartile ranges above the 75th percentile of the individual RT distribution was regarded as an outlier (Tukey, 1977).

^2^
Note that we ran two other experiments where we tested the indirect response activation effect among various classes of S1–S2 associations such as adjective pairs or trait-name pairs. Indirect retrieval effects were absent for arbitrary linked words (adjective word pairs) and weak but significant for adjective-trait word pairs. Material, data, and analyses for these unpublished data are accessible at the OSF repository (https://osf.io/aj2eg/).

^3^
We want to thank an anonymous reviewer for making us aware of this alternative account.

## Notes

**Publication Ethics:** Ethical approval was not required for this study as we did not convey any misleading or suggestive information (this is in accordance with the ethical standards at the Institute of Psychology of FSU Jena).

**Authorship:** Mrudula Arunkumar, conceptualization, data collection & analysis, writing – original draft preparation, writing – review & editing; Klaus Rothermund, writing – review & editing, supervision; Carina G. Giesen, conceptualization, writing – review and editing, supervision.

**Open Data:** To the best of my ability and knowledge, I have provided all original materials and clear references to all other materials via a stable online repository.

The study design and analyses plan for Experiment 1a and Experiment 1b were preregistered on the Open Science Foundation (OSF) using the AsPredicted.com template (https://doi.org/10.17605/OSF.IO/FC5U3, Arunkumar et al., 2021, for Experiment 1a and https://doi.org/10.17605/OSF.IO/TCRM5, Arunkumar et al., 2023a, for Experiment 1b).

The open repository containing analysis scripts and materials for both the experiments is as follows: https://osf.io/aj2eg/ (Arunkumar et al., 2023b).

The OSF repository also contains the data file for preliminary experiments that were performed and referred to in Footnote 2.
